# Purification and Characterization of Tannin Acyl Hydrolase Produced by Mixed Solid State Fermentation of Wheat Bran and Marigold Flower by *Penicillium notatum* NCIM 923

**DOI:** 10.1155/2013/596380

**Published:** 2013-11-17

**Authors:** Saswati Gayen, Uma Ghosh

**Affiliations:** ^1^Department of Microbiology, Vijaygarh Jyotish Ray College, Kolkata 700032, West Bengal, India; ^2^Department of Food Technology and Biochemical Engineering, Jadavpur University, Kolkata 700032, West Bengal, India

## Abstract

Tannin acyl hydrolase produced extracellularly by the fungal strain *Penicillium notatum* NCIM 923 in mixed solid state fermentation of wheat bran and marigold flower in the ratio 4 : 1 was purified from the cell-free extract broth by ammonium sulphate fractionation followed by diethylaminoethyl-cellulose column chromatography. Tannase was purified by 19.89-fold with yield of 11.77%. The specific activity of crude tannase was found to be 1.31 U/mg protein while that of purified tannase was 22.48 U/mg protein. SDS-PAGE analysis indicated that the enzyme is dimeric with one major band of molecular mass 97 kDa and a very light band of molecular mass 43 kDa. Temperature of 35 to 40°C and pH 5 were optimum for tannase activity. The enzyme retained more than 60% of its stability at 60°C and 40% stability at pH 3 and 8, respectively. *K*_*m*_ was found to be 0.33 × 10^−2^ M and *V*_max_ = 40 U/mg. Since the enzyme is active over a wide range of pH and temperature, it could find potential use in the food processing industry.

## 1. Introduction

Tannins are phenolic compounds, which can be grouped as hydrolysable and nonhydrolyzable tannins. Tannin acyl hydrolase (E.C.3.1.20), commonly named tannase, only hydrolyses the hydrolysable tannins and catalyses the hydrolysis of ester and depside bonds in tannic acid releasing glucose and gallic acid [1]. It has extensive applications in the food, feed, pharmaceutical, beverage, and chemical industries and is extensively used in wine, beer, and coffee-flavored soft drinks or as an additive in food detannification [2]. The enzyme can be used to reduce the concentration of tannic acid in tannery effluent thereby reducing the pollution level of tannery wastewater [3]. Gallic acid, the product of the reaction, is used in the enzymatic synthesis of propyl gallate, which is mainly used as an antioxidant in fats and oils [4, 5]. 

 Tannase production can be achieved by various methods such as liquid surface, submerged (SmF), and solid state fermentation [1]. Tannase production by solid state fermentation (SSF) is more advantageous as compared to submerged or liquid surface fermentation [6]. Filamentous fungi are ideally suited for successful SSF since they grow on solid substrate in the absence of free water [2]. SSF offers numerous opportunities in utilizing agricultural and floricultural residues to value added products. The use of byproducts or residues rich in carbon sources for growth and fermentation purposes is an alternative to solve pollution problems caused by incorrect disposal of agricultural and horticultural wastes [7].

 Purification and characterization of tannase had been attempted earlier from plant and microbial sources [8]. Usually the end products of a fermentation process contain some unwanted components, which have to be eliminated as far as possible by downstream processing [2]. The purification and characterization of tannase had been attempted owing to its applications in various food, feed, leather, and pharmaceutical industries. Techniques such as affinity chromatography, ultrafiltration, high performance liquid chromatography, and electrophoresis. are used to attain high level of purification. However these processes are expensive and result in low yields [2]. Thus the purified enzymes lose their activity and become unsuitable for biochemical studies. But preservation of function is required for activity studies. For commercial applications, extremely high levels of enzyme purity are not required and a quick purification procedure is necessary to keep the process inexpensive [7]. Thus it was considered significant to purify and characterize the pure enzyme to explore the factors affecting their activity. The present study describes purification and characterization of tannase produced by *Penicillium notatum* NCIM 923 whose biochemical properties may render it of commercial interest.

## 2. Materials and Methods

### 2.1. Microorganism and Maintenance of Culture

*Penicillium notatum* NCIM 923 was collected from National Collection of Industrial Microorganisms (NCIM), National Chemical Laboratory, Pune (India), and was maintained on Czapek Dox agar slants of composition—Glucose 5%, NaNO_3_ 0.2%, KCl 0.05%, MgSO_4_·7H_2_O 0.05%, FeSO_4_·7H_2_O 0.001%, KH_2_PO_4_ 0.1%, Agar 3% pH 5. The fungal strains were cultured periodically, grown at 30°C for 6 days, and were stored at 4°C.

### 2.2. Production of Tannase Under SSF

Fermentation medium used for tannase production contained 10 g of substrate (wheat bran and marigold flower mixed in the ratio 4 : 1) [9] in a 500 mL Erlenmeyer flask moistened with (1 : 1 w/v) distilled water. The C : N ratio of the mixed substrate was found to be 1.26. The sterilized (15 psig, 15 mins) solid substrate was inoculated with spore suspension (1 × 10^7^ spores/mL). The contents were mixed properly and incubated at 30°C for 96 hrs under stationary condition [9]. 

### 2.3. Extraction and Analysis of Crude Enzyme

Mouldy substrate produced by SSF was mixed with distilled water (1 : 5 w/v) and agitated for 2 hrs at 90 rpm in a shaker incubator and filtered through cheese cloth followed by centrifugation at 10,000 rpm for 20 mins. The clear supernatant was used as crude enzyme.

 Tannase activity was estimated by a protein precipitation method [10]. The reaction mixture contained 1 mL 1% tannic acid (in citrate phosphate buffer, pH 5.0), 2 mL of citrate phosphate buffer (pH 5.0) and 1 mL of the culture filtrate. The mixture was incubated at 37°C for 30 mins in a water bath. The reaction was stopped by adding 4 mL 2% BSA solution. In the control BSA was added in the incubation mixture prior to incubation. All tubes were left for 20 mins at room temperature to precipitate residual tannins and were centrifuged at 3000 ×g for 20 mins. The tannase activity in the supernatant was estimated after appropriate dilution and reading O.D. at 260 nm (this wavelength corresponds to the optimal absorption of gallic acid) against double distilled water as blank. One enzyme unit is the amount of enzyme that liberates 1 *μ* mol gallic acid per mL per min under standard assay conditions.

 Total soluble protein was determined by the method of (Lowry et al., [11]) and was expressed in mg/mL.

### 2.4. Purification and Characterization of Tannase

#### 2.4.1. Ammonium Sulphate Fractionation and Dialysis

The crude culture filtrate obtained after centrifugation was concentrated 3 times using rotary vacuum evaporator (EYELA Rotary Evaporator N 1000, Japan). Ammonium sulphate precipitation was done according to Englard and Scifter [12]. Ammonium sulphate required to precipitate tannase was optimized by its addition, at varying levels of concentrations (10–100% saturation), to the concentrated crude extract. Precipitated protein was collected by centrifugation at 10,000 rpm for 15 mins at 4°C. The precipitate was resuspended in 10 mL 0.1 M citrate phosphate buffer (pH 5). The enzyme activity and protein content of the fractions were measured. This precipitation process was continued up to 100% ammonium sulphate saturation. The fraction showing maximum specific activity was used for further purification.

 The precipitate obtained after ammonium sulphate precipitation was further dialyzed against the 0.1 M citrate phosphate buffer (pH 5) in order to remove the ammonium sulphate from the precipitate. Dialysis was carried out for 24 hrs with several changes of 0.1 M citrate phosphate buffer (pH 5). 

#### 2.4.2. DEAE-Cellulose Chromatography

10 mL of the dialysate was applied to the previously equilibrated DEAE-cellulose column. The enzyme was eluted with 0.1 M citrate phosphate buffer (pH 5) containing a linear gradient of 0–2 M NaCl at a flow rate of 20 mL/h. Eluted fractions (5 mL) were analyzed for protein content and tannase activity. Active fractions were freeze-dried and stored at 4°C for further studies. Purification fold and enzyme yield were determined.

#### 2.4.3. Molecular Weight Determination

Molecular mass of purified enzyme was determined by sodium dodecyl sulphate-polyacrylamide gel electrophoresis (SDS-PAGE) according to the method of Laemmli's [13]. Electrophoresis was done on 12% gel and the separated protein band was detected by Coomassie blue staining. The stacking gel consisted of 5% polyacrylamide. Molecular mass markers were purchased from Bangalore Genei (India) and run parallel to the samples.

#### 2.4.4. Temperature Activity Profile

To determine the temperature activity profile the enzyme substrate reaction was carried out at various temperatures (30–60)°C using the crude as well as purified enzyme in 0.1 M citrate phosphate buffer (pH 5) for 30 mins with tannic acid as substrate and enzyme activity was measured [14].

#### 2.4.5. Thermal Stability

Thermostability of the crude and purified enzyme was determined by 60 mins incubation over the temperature range (30–60)°C at (pH 5). Residual activity was estimated under standard conditions and expressed as percentage of the relative tannase activity [15].

#### 2.4.6. pH Activity Profile

The pH activity profile for crude and purified tannase was determined at 30°C by incubating the enzyme with substrate at different pH ranges from 3 to 8. The pH of the reaction mixture was varied using different buffers (0.1 M citrate phosphate buffer for pH 3–7 and tris HCl buffer for pH 8) and enzyme activity was measured [14]. 

#### 2.4.7. pH Stability

The stability of the crude and purified enzyme was examined at different pH values by incubating the enzyme in buffers at different pH values ranging from 3 to 8 (0.1 M citrate phosphate buffer for pH 3–7 and tris HCl buffer for pH 8) for 12 hrs at 30°C. Residual activity was estimated under standard conditions and expressed as percentage of the relative tannase activity [16].

#### 2.4.8. Kinetic Parameters

The effect of substrate concentration on the activity of the crude and purified tannase was determined by using different concentrations of tannic acid (0.5, 0.8, 1, 1.5, and 2) %, in the reaction mixture and the enzyme activity was estimated in each case. *K*_*m*_ and *V*_max⁡  _were determined by plotting the reaction velocity against the substrate-tannic acid concentration in the Lineweaver-Burk plot [17]. 

 All the experiments were done in triplicate and the mean values with standard errors are reported.

## 3. Results and Discussion

### 3.1. Purification of Tannase

Tannase was produced by mixed solid state fermentation of wheat bran and used marigold flower in the ratio 4 : 1 by *P. notatum* NCIM 923. Enzyme purification was achieved by a combination of concentration by rotary vacuum evaporator followed by ammonium sulphate precipitation and chromatographic resolution using DEAE-cellulose column. Initially the 3-time concentrated enzyme was purified by precipitation with ammonium sulphate at the saturation level of 75%. In this step, the enzyme was purified 5.96-fold with a yield of about 65.72% and the specific activity was 6.74 U/mg protein. After dialysis of the 75% fraction a specific activity of 10.40 U/mg protein was obtained with 9.20-fold purification (Table 1). Rajkumar and Nandy obtained a yield of 69% with ammonium sulphate precipitation [18]. Hamdy reported that 65% ammonium sulphate was optimum for tannase precipitation [19]. Sabu et al. [20] found 4-fold purification yield of the dialyzed tannase with specific activity of 0.916 U/mg protein. Paranthaman et al., [21] reported maximum tannase activity at 60% ammonium sulphate fraction.

**Figure 1:**
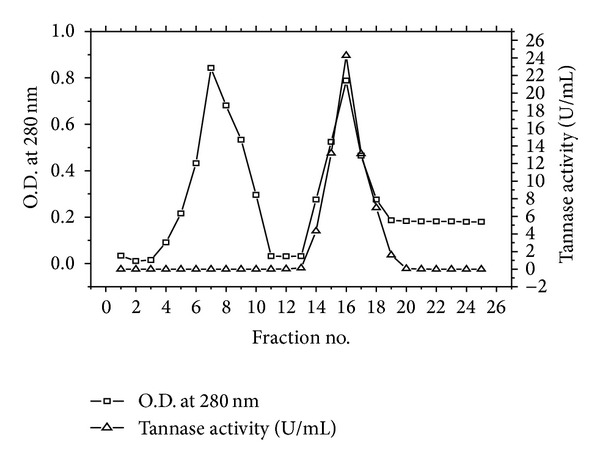
Elution profiles of tannase from *P. notatum* NCIM 923 in DEAE-cellulose column chromatography.

 The dialyzed sample was further purified by ion exchange chromatography using DEAE-cellulose column equilibrated with 0.1 M citrate phosphate buffer (pH 5). The proteins were eluted with a NaCl gradient (0–2 M). The elution profile is shown in Figure 1. Most of the proteins were present in two major peaks. However, tannase activity was detected only in the 2nd fraction (Figure 1). By DEAE-cellulose column chromatography, 19.89-fold purification of the enzyme was achieved and its specific activity was found to be 22.48 U/mg protein with a yield of 11.77% (Table 1). Similar observations of 19.5-fold and 19.4-fold purification of tannase using DEAE-cellulose column chromatography were reported by Chhokar et al. [22] and Mahendran et al. [23], respectively.

### 3.2. Molecular Mass Determination of Tannase

**Figure 2:**
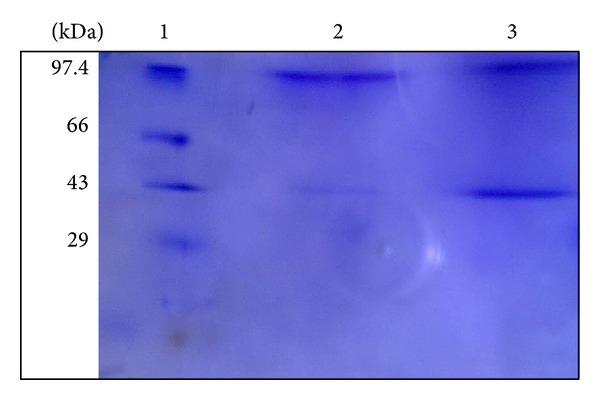
Sodium dodecyl sulphate-polyacrylamide gel electrophoresis (SDS-PAGE) of purified tannase from *Penicillium notatum* NCIM 923. The lanes contain (1) molecular weight markers, (2) bands corresponding to a molecular mass of approximately 97 kDa (major) and 43 kDa (minor) from purified sample, and (3) bands corresponding to 97 kDa and 43 kDa (approx.) from partially purified sample.

The molecular mass of the purified enzyme was determined by SDS-PAGE (Figure 2). The purified enzyme showed two bands—one major band of molecular mass of about 97 kDa and a very light band of molecular mass of about 43 kDa. Most of the purified tannase from filamentous fungi has a total molecular mass in the range of 168 to 310 kDa [24, 25]. Tannases have generally high molecular weight and multimeric forms. The tannase from *Paecilomyces variotii* had a major band of 87.3 kDa and a minor band of 71.5 kDa [16]. Tannase from *Verticillium *sp. P9 had two kinds of subunits with molecular masses of 39.9 and 45.6 kDa [15]. According to Hatamoto et al. the tannase from *A. oryzae* has two subunits of 30 and 33 kDa [26]. The molecular mass of *A. niger* MTCC 2425 tannase has been reported to be 185 kDa with two polypeptide chains of apparent molecular masses of 102 and 83 kDa [27]. 

### 3.3. Properties of Tannase

#### 3.3.1. Temperature Optima for Tannase Activity

**Figure 3:**
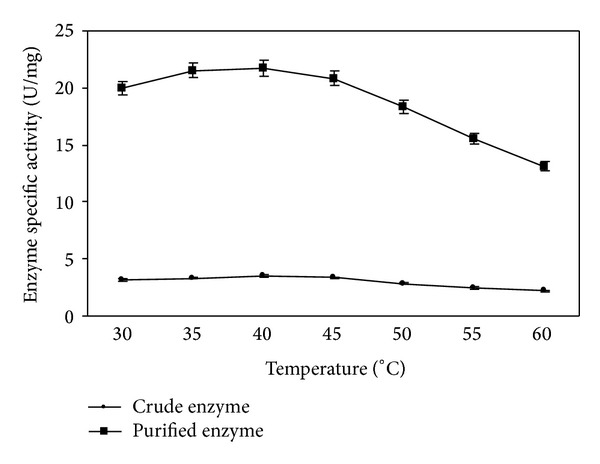
Effect of temperature on tannase activity.

Figure 3 represents the temperature optima of both crude and purified tannase. The enzyme was active in the temperature range of 30–60°C with an optimal activity (21.72 U/mg) at 40°C. Sivashanmugam and Jayaraman also reported optimal activity of tannase from *K. pneumoniae* MTCC 7162 at 40°C [14]. Sabu et al. reported that optimal activity of tannase from *A. niger* ATCC 16620 was in the range 30–40°C [20]. Similar observations were reported for tannase from *A. oryzae*, *Aspergillus* sp., and *P. chrysogenum* [18, 28, 29]. Further increase in temperature tannase activity was found to decrease for both crude and purified enzyme. This may be due to the fact that increase in temperature increases the rate of denaturation of the enzyme, with the loss of its structure. The purified enzyme was fairly active even at 60°C (13.11 U/mg) and this can be considered as an additional advantage, since most of the processes assisted by tannase are performed at increased temperatures [20].

#### 3.3.2. Thermal Stability of Tannase

**Figure 4:**
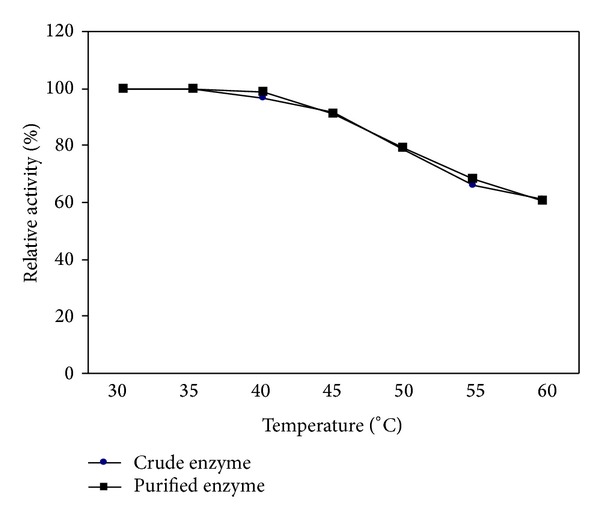
Effect of temperature on stability of tannase enzyme.

The thermal stability profile of both crude and purified tannase (Figure 4) revealed that above 95% was retained at 40°C which was found to be the optimal temperature for tannase activity (Figure 3). Sivashanmugam and Jayaraman reported that the thermal stability of tannase from *K. pneumoniae* MTCC 7162 was retained by more than 50% at 60°C [14]. Above 40°C both enzymes began to lose the thermal stability but even at 60°C more than 60% of thermal stability was retained by both of the enzymes. This result indicates that extracellular tannase produced by *P. notatum* NCIM 923 is active over a wide range of temperature. An increase of temperature beyond the optimum value showed a decrease in the catalytic rate of tannase. This may be due to the fact that either the enzyme or substrate became denatured, resulting in catalytic inactivation of the enzyme. Temperatures above the optimum value also affect the protein ionization state and the solubility of species in solution which resulted in a reduction in enzyme activity [2]. 

#### 3.3.3. pH Optima for Tannase Activity

**Figure 5:**
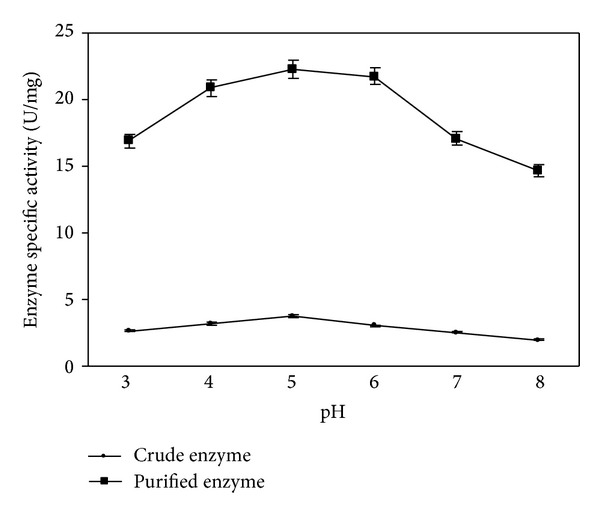
Effect of pH on tannase activity.

The pH optima for both crude and purified tannase were pH 5 (Figure 5). The enzymes were found active in the pH range of 3–8 with an optimal activity at pH 5 (22.27 U/mg). Below or above pH 5 enzyme activities decreased for both crude and purified. Tannase from *K. pneumoniae* MTCC  7162 showed pH optima at 5.5 [14], for *Paecilomyces variotii* optimal pH was reported 5–7 [23], and pH 5 was reported for *P. chrysogenum* [19]. The effect of pH on the enzyme activity depends on the nature of ionization of amino acids at the active site. It is also influenced by the conformational changes resulted from the ionization of other amino acids. Enzymes are very sensitive to changes in pH and they function best over a very limited range, with a definite pH optimum [20].

#### 3.3.4. pH Stability of Tannase

**Figure 6:**
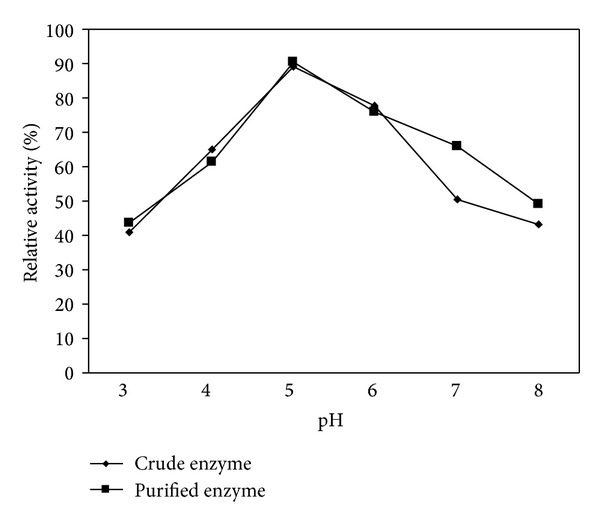
Effect of pH on stability of tannase enzyme.

The pH stability profile of both crude and purified tannase is shown in Figure 6. The crude and purified tannase showed the same tendencies for pH stability. Below or above pH 5 the pH stability was found to decrease. A stability of above 85% was retained by both the enzymes even after 12 hrs of incubation. Even at pH 3 and 8 the enzymes exhibited more than 40% of activity with respect to the activity of optimal pH after 12 hrs of incubation. Sivashanmugam and Jayaraman reported a pH stability of 81.3% at pH 5 of tannase from *K. pneumoniae* MTCC 7162 [14].

#### 3.3.5. Kinetic Parameters

**Figure 7:**
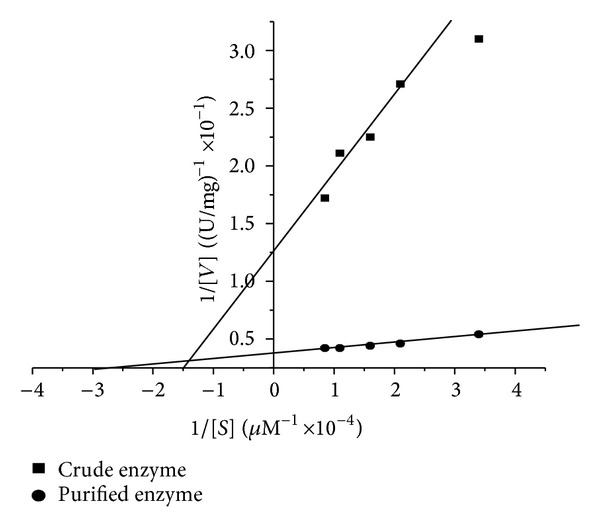
Kinetic parameters of crude and purified tannase.

The effect of substrate concentration on tannase activity was studied with various concentrations of tannic acid. The analysis of the graph of substrate concentration against tannase activity (Figure 7) yielded values for *K*_*m*_ of 0.33 × 10^−2 ^M and *V*_max⁡  _ of 40 U/mg for the purified enzyme. The crude enzyme showed *K*_*m*_ value 0.66 × 10^−2 ^M of and *V*_max⁡  _ value of 8.0 U/mg. Purified tannase from *P. chrysogenum *was reported to have a *K*_*m*_ value of 0.48 × 10^−4 ^M [18]. Tannase from *Cryphonectria parasitica* showed a *K*_*m*_ value of 7.49 mM [30]. Nadaf and Ghosh observed that the *K*_*m*_ of tannase I was 0.034 mM, whereas the *K*_*m*_ of tannase II was 0.040 mM. The *V*_max⁡  _ of both enzymes (isolated from *Rhodococcus* NCIM 2891) was found to be 40 and 45 U/mL, respectively [31].

## 4. Conclusion

The present study reports the purification of tannin acyl hydrolase produced by utilization of wheat bran used marigold flower with *P. notatum* NCIM 923. The purified enzyme was active over a wide range of pH and temperature. Furthermore the purified enzyme was active with more than 60% of its activity even at 60°C. The other quality of the enzyme was its low *K*_*m*_ value. All these characteristics are considered to be promising for applications in food processing industry.

## Figures and Tables

**Table 1:** Purification of tannase from *P.  notatum* NCIM 923.

Purification stage	Total activity (U)	Total protein (mg)	Specific activity (U/mg)	Purification fold	Yield (%)
Crude extract	2067	1814	1.13	1	100
Concentrated	1733	676	2.56	2.26	83.84
75% ammonium sulphate saturated	1358.5	201.5	6.74	5.96	65.72
Dialyzed sample	267.8	25.75	10.40	9.20	12.96
DEAE cellulose column chromatography	242.8	10.8	22.48	19.89	11.77
